# Tapinarof cream 1% for corticosteroid-refractory erythema annulare centrifugum: A first reported case

**DOI:** 10.1016/j.jdcr.2026.05.052

**Published:** 2026-05-26

**Authors:** Hamza Malick, Scott Jaros, Kritin K. Verma, Dario Kivelevitch

**Affiliations:** a Division of Dermatology, Baylor University Medical Center, Dallas, Texas; b Texas Tech University Health Sciences Center, School of Medicine, Lubbock, Texas; c Clarity Dermatology, Dallas, Texas

**Keywords:** annular plaque, aryl hydrocarbon receptor, erythema annulare centrifugum, figurate erythema, refractory dermatosis, steroid-sparing therapy, tapinarof

## Introduction

Erythema annulare centrifugum (EAC) is a rare reactive figurate erythema that is defined by slowly growing annular or polycyclic erythematous plaques with a distinctive trailing scale and central clearing.^1^ The etiology is thought to be a T cell-mediated hypersensitivity reaction to a variety of antigenic triggers, such as infections, autoimmune disorders, cancer, and drugs, the latter of which includes metformin as a known cutaneous trigger.^1^ Histopathologically, the superficial variant demonstrates a well-demarcated, “coat-sleeve” perivascular infiltrate of lymphocytes and histiocytes within the superficial vascular plexus, often accompanied by papillary dermal edema and limited epidermal changes, whereas the deep variant is characterized by a predominantly mononuclear perivascular infiltrate involving the mid to lower dermis with a largely unremarkable epidermis and more indurated clinical morphology.^1^

Topical corticosteroids, though used first-line, yield inconsistent and often transient results, and prolonged use carries well-established risks, including cutaneous atrophy. Emerging case reports documenting responses to upadacitinib, roflumilast, and apremilast support targeted immunomodulation as a viable strategy in corticosteroid-refractory disease.^2^, ^3^, ^4^ We report the first case of EAC successfully treated with tapinarof cream 1%, a nonsteroidal topical aryl hydrocarbon receptor (AhR) agonist.

## Case report

A 61-year-old woman with Fitzpatrick skin type III presented with a 4-year history of a pruritic erythematous eruption involving the bilateral upper extremities and left lower extremity. The patient denied blisters, fever, chills, sore throat, diarrhea, cough, and arthralgias. The eruption started temporally after she switched from immediate-release to extended-release metformin for her antidiabetic prescription, which raised the possibility of a medication trigger. Nevertheless, metformin was continued because it was still clinically necessary. Mometasone furoate 0.1% cream, administered twice a day in the past, was not able to give sufficient control for an extended period.

**Fig 1:**
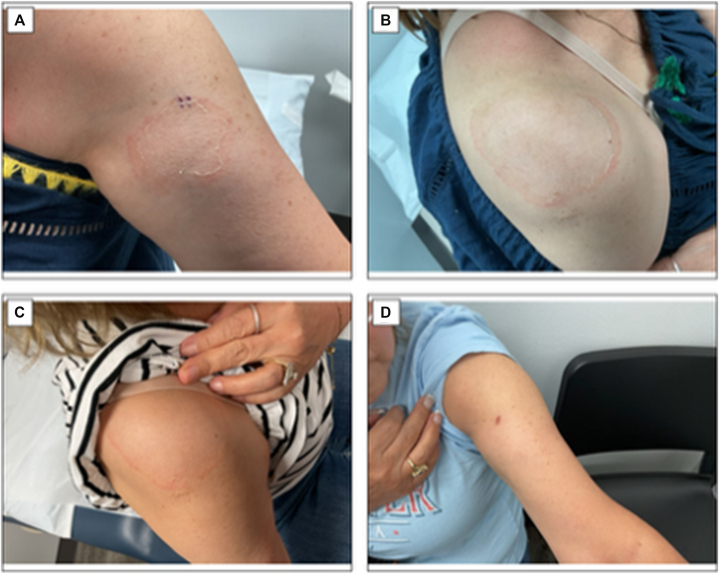
**A,** Baseline close-up of the right proximal upper arm demonstrating an annular erythematous plaque with trailing scale and biopsy site. **B,** Baseline wide-field view confirming the annular configuration. **C,** One week after initiating tapinarof cream 1% once daily, showing partial regression with reduced erythema and scale. **D,** Complete clearance at follow-up with residual biopsy scar; no active erythema, scale, or recurrence.

Upon physical examination, the right proximal upper arm showed a clearly defined annular erythematous plaque with scale at the inner advancing edge (Fig 1, *A* and *B*). The lesion was nontender, nonindurated, and slightly to moderately inflamed. The patient had no other erythematous or pruritic lesions to report elsewhere on the body, including the feet, axilla, and groin.

A 4-mm punch biopsy was taken from the lesion's active border. Under a microscope, the dermis showed a sparse superficial perivascular lymphocytic infiltration, focal mounds of parakeratosis, dispersed neutrophils, and an acanthotic epidermis with compact keratin. Although there was little evidence of classic extensive perivascular “coat-sleeve” cuffing, direct clinicopathologic correlation was most compatible with the superficial variety of EAC. Fungal hyphae were not detected by periodic acid–Schiff staining, ruling out tinea as a possible alternative diagnosis.^5^

Following biopsy-confirmed EAC and failure of topical corticosteroid therapy, mometasone was discontinued and tapinarof cream 1% once daily was initiated. Clinical photos showed significant partial regression with decreased scale and erythema at 1 week (Fig 1, *C*). At 4-week follow-up, the eruption had entirely subsided, leaving just the biopsy scar (Fig 1, *D*). Throughout the course of treatment, the patient did not report any side effects. Two months after stopping treatment with tapinarof the patient had no signs of recurrence.

## Discussion

The pathophysiology of EAC is characterized by perivascular inflammation that is dominated by CD4+ and CD8+ T lymphocytes and is caused by delayed-type hypersensitivity to a variety of antigens.^6^ A proposed Th1-mediated component, characterized by elevated TNF-α and proinflammatory cytokines, may further sustain perivascular inflammation and contribute to the incomplete response observed in corticosteroid monotherapy.^6^ The temporal association between the introduction of extended-release metformin and the onset of EAC in this case is consistent with previous published reports of metformin-induced urate erythema.^2^, ^3^, ^4^^,^^6^ The independent contribution of tapinarof to clearance cannot be completely separated from possible spontaneous remission because metformin was medically required and continued throughout treatment. Nonetheless, the 4-year refractory course greatly reduces the likelihood of coincidental resolution.

Although topical corticosteroids remain the most commonly used first-line treatment for EAC, achieving complete, long-lasting response rates is difficult, and prolonged use may carry the risk of telangiectasias and cutaneous atrophy.^1^^,^^7^ Published cases demonstrating clearance with upadacitinib (selective JAK1 inhibition),^2^ roflumilast (topical PDE4 inhibition),^3^ and apremilast (PDE4 inhibition)^4^ collectively support the idea that cytokine-directed therapy is an effective and durable strategy for refractory disease.^1^^,^^6^

Tapinarof is a naturally occurring stilbenoid and first-in-class nonsteroidal topical AhR agonist.^8^ AhR is a ligand-dependent transcription factor constitutively expressed in keratinocytes, dendritic cells, and T lymphocytes that governs epidermal barrier integrity and cutaneous immune homeostasis.^6^^,^^8^ AhR undergoes nuclear translocation upon binding tapinarof, which results in the downstream inhibition of proinflammatory cytokines, including IL-4, IL-13, IL-17A, and TNF-α, while concurrently upregulating barrier proteins filaggrin, loricrin, and involucrin.^6^^,^^8^^,^^9^ This dual immunomodulatory and barrier-restorative mechanism is mechanistically well-suited for EAC, in which T cell–mediated perivascular inflammation and impaired barrier function sustain ongoing antigen permeability and immune activation.

Rapid partial regression within 7 days and complete clearance at subsequent follow-up, despite a 4-year corticosteroid-refractory course, supports tapinarof as an effective steroid-sparing option in refractory EAC. Notably, our patient did not experience folliculitis, or other adverse effects, consistent with tapinarof’s favorable safety profile.^8^^,^^9^ Because of its nonsteroidal mechanism of action, acceptable safety profile, and topical application, tapinarof may be well suited for patients who need long-term treatment, have disease on steroid-sensitive anatomic locations, or are not candidates for systemic immunomodulators.^6^^,^^8^^,^^9^ To our knowledge this represents the first reported case of EAC treated with tapinarof. Further reports are warranted to establish optimal treatment duration, durability of remission following discontinuation.

### Declaration of generative AI and AI-assisted technologies in the writing process

No artificial intelligence (AI) or large language models were used in the writing, data analysis, figure/table creation, or editing of this manuscript. All text and analyses were conducted by the authors.

## Conflicts of interest

None disclosed.
